# Glucose–insulin–potassium therapy in patients with acute coronary syndrome: a meta-analysis of randomized controlled trials

**DOI:** 10.1186/1471-2261-14-169

**Published:** 2014-11-25

**Authors:** Pei-Yin Jin, Hai-San Zhang, Xiao-Yan Guo, Wei-Fang Liang, Qin-Fu Han

**Affiliations:** Department of Cardiovascular Medicine, The People’s Hospital of Anyang, Anyang, Henan China; The Second Affiliated Hospital of Xinxiang Medical University, Xinxiang, 453002 Henan China

**Keywords:** Glucose–insulin–potassium, Acute coronary syndrome, Meta-analysis

## Abstract

**Background:**

Glucose-insulin-potassium (GIK) has been advocated in the setting of acute coronary syndrome (ACS) to reduce ischemia-related arrhythmias and myocardial injury. We conducted a meta-analysis of randomized controlled trials (RCTs) to assess whether the use of GIK infusions >3 or <3 hours after the onset of symptoms reduce mortality or cardiac arrest.

**Methods:**

Electronic databases (Medline, EMBASE, and Cochrane Central Register of Controlled Trials) and references of retrieved articles were searched for RCTs evaluating the effect of GIK infusions, <3 hours or >3 hours after the onset of symptoms, on mortality and/or cardiac arrest. Pooled odds ratios (ORs) with 95% confidence intervals (CIs) were calculated for each outcome.

**Results:**

Nine trials were identified and eligible for review. The summary OR for in-hospital mortality was 1.01 (95% CI 0.94 to 1.09), based on 2,542 deaths among 27,294 patients. The subgroup analysis according to the study enrollment time (within 3 hours [OR, 0.77, 95% CI 0.50-1.16], vs. >3 hours [OR, 0.90; 95% CI, 0.67-1.21]) did not reveal any difference in mortality.

**Conclusions:**

Administration of GIK in ACS patients does not significantly reduce mortality whether or not GIK administration >3 or <3 hours after the onset of symptoms.

**Electronic supplementary material:**

The online version of this article (doi:10.1186/1471-2261-14-169) contains supplementary material, which is available to authorized users.

## Background

Glucose–insulin–potassium (GIK) has long been advocated as an adjunctive treatment for patients with cardiac dysfunction during episodes of ischemia and reperfusion. Experimental and clinical studies have demonstrated that GIK could improve the efficiency of energy use, reduce circulating free fatty acids, and be anti-apoptotic [1, 2]. GIK has now been commonly applied in patients with acute coronary syndrome (ACS) as a metabolic support to ischemic myocardium.

Since the first application of GIK in the setting of cardiac ischemic disease in the 1962 [3], GIK therapy has gone through alternating periods of varying attention. Early trials in the setting of acute myocardial infarction (AMI) suggested a benefit from GIK therapy (6). However, a recent large, randomized, placebo-controlled trial [4] has failed to show any survival benefit, even revealed increased morbidity. A 2010 meta-analysis of eight randomized trials that involved >22,000 patients did not reveal any mortality benefit with GIK therapy in ST-segment–elevation AMI. However, all the studies examined AMI patients ≥3 hours after the onset of symptoms except for Glucose-Insulin- Potassium Study-1 (GIPS-1), which accounted for only 3% of the total population in the meta-analysis [4]. The most recent completed IMMEDIATE trial [5]. Their hypothesis was that the timing of GIK-infusion was responsible for the inconsistent results of prior GIK-trials. Therefore, 871 patients with a suspected acute coronary syndrome (ACS) were randomized to GIK infusion or placebo in the ambulance, thereby significantly shortening system delay. So there is still uncertainty regarding GIK clinical effectiveness according to the study enrollment time (within 3 hours vs. >3 hours). The objective of this analysis was to systematically review randomized trials to assess the effectiveness of GIK in ACS patients.

## Methods

We followed the preferred reporting items for systematic reviews and meta-analyses guidelines (PRISMA) to report our study findings [6].

### Eligibility criteria

The study’s eligibility criteria were as follows: (1) RCT, (2) comparison of GIK as adjunctive therapy, (3) report of a risk estimate (relative risk, odds ratio, or data from which it could be calculated), and (4) report of all-cause mortality.

### Data sources and search strategies

The PubMed (1966 to May 2014), EMBASE (1966 to May 2014) and the Cochrane Central Register of Controlled Trials (CENTRAL, 1996 to May 2014) were searched for randomized, placebo controlled trials that examined the adjunctive use of GIK in the setting of ACS using the Cochrane randomized controlled trial filter and the following MeSH headings/text words: coronary artery disease, acute coronary syndrome, myocardial infarction, AMI, ACS, glucose-insulin-potassium and GIK Electronic searches were supplemented with a review of the reference lists of retrieved articles and by contacting experts in the field. The electronic search was up to date as of May 2014 and no language restrictions were applied.

### Data extraction

Two individuals (Wei-Fang Liang and Qin-Fu Han) independently extracted data from eligible articles. Data extracted included GIK protocol (dose and infusion method), demographic data, trial characteristics, outcome data (all-cause mortality).

### Quality assessment

We evaluated the quality of the evidence by using the GRADE (Grades of Recommendation, Assessment, Development and Evaluation) approach [7, 8]. In addition, the GRADE profiler 3.6 software was used to create the evidence profile. GRADE Working Group grades of evidence were as follows: High quality: Further research is very unlikely to change our confidence in the estimate of effect. Moderate quality: Further research is likely to have an important impact on our confidence in the estimate of effect and may change the estimate. Low quality: Further research is very likely to have an important impact on our confidence in the estimate of effect and is likely to change the estimate. Very low quality: We are very uncertain about the estimate.

### Subgroup analysis

Subgroup analyses of the time of GIK administration (before or after reperfusion) were performed because Dr. Opie suggests that GIK infusion in A MI is more likely to be beneficial when given before reperfusion therapy [9]. Further subgroup analyses of studies comparing GIK with standard therapy were performed according to duration of therapy from AMI onset (<3 hours and >3 hours) [2].

### Statistical analyses

Categorical variables were reported as frequency and proportions. Relative estimate was presented by using odd ratios (ORs) with 95% CI, calculated by using the fixed effects model. The heterogeneity among the studies was assessed by using the I^2^ statistic and the Cochran Q statistic for each outcome [10]. A P value of less than 0.10 of the Cochran Q test suggests that the heterogeneity is beyond random error or chance [10]. Meta-regression was performed to assess the influence of duration of therapy from AMI onset on the pooled estimate of effect. P values were considered significant for P < 0.05. Statistical analyses were performed using Review Manager (RevMan) Version 5.1 and STATA (StataCorp LP, USA) version 11.0.

### Ethics

This meta analysis didn’t require ethical approval.

## Results

### Identification of eligible studies

**Figure 1:**
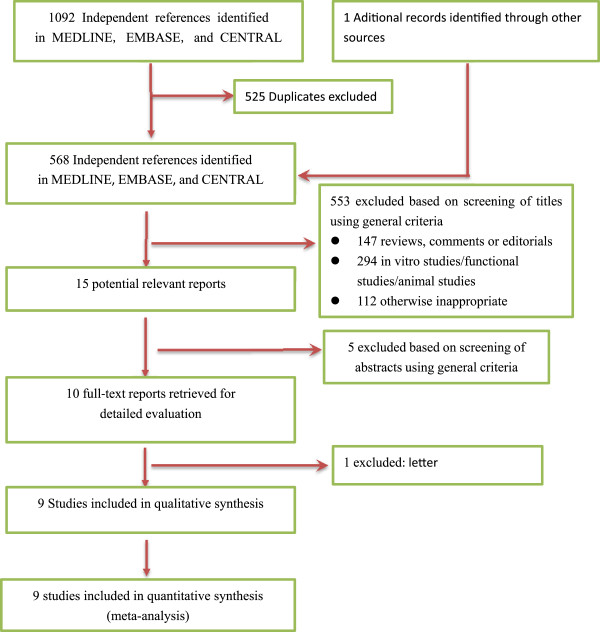
**Identification of eligible studies.**

A total of 567 unique records were identified through comprehensive database search, and 1 additional article was identified from other sources. Of these citations, nine trials [4, 5, 11–17] met the inclusion criteria in our meta-analysis (see Figure 1 for the preferred reporting items for systematic reviews and meta-analyses guidelines (PRISMA) flow diagram).

### Characteristics of eligible studies

The studies used different perioperative GIK protocols. Study protocols and participant characteristics are presented in Table 1. Three trials did not report time from onset of symptom to treatment [14, 16, 17]. Two trials reported time from onset of symptom to treatment within 3 hours [5, 13], while four trials reported time from onset of symptom to treatment >3 hours [4, 11, 12, 15]. No included trials were at low quality, and the GRADE assessment of strength of evidence varied from moderate to high quality (Additional file 1) because of unclear reporting of allocation concealment and blinding in many studies.

**Table 1:** **Characteristics of included studies**

Study	No. of patients	Age*	Male(%)*	Killip I (%)*	Diabetes (%)*	Glucose (%)	Insulin (U/500 ml)	Potassium (%)	Infusion rate (ml/kg/h)	Time from onset of symptom to treatment (h)*	24-h glucose (mmol/L)*
ECLA (1998))	470	58.2/60.5	77/68.3	84.4/86.3	18.5/18	25	50	0.3	1.5	11.4/10.6	4.25/4.04
POL-GIK (1999)	954	62/60	70/67.2	94.1/97	6.5/6.1	10	32	0.3	42 ml/h	5	5.9/6.2
GIPS-I (2003)	940	59.9/60.8	73.7/79.3	89.5/92.7	10.5/10.6	20	7-11	0.3	3	2.75-2.78/2.75-2.82	7.7/8.1*
REVIAL (2004)	312	60.8/64.1	71.6/72.6	67.7/69.4	22.6/23.6	20	40	0.25	1.8	-	-
CREATE-ECLA (2005)	20201	58.6/58.6	77.6/77.6	84.2/85.1	17.6/17.8	25	50	0.3	1.5	4.7/4.7¶	8.6/7.5
Krljanac G (2005)	118	56.6/56.7	66.7/72.5	-	17/17	25	50	0.3	1	3.1/3.2	-
GIPS-II (2006)	889	61.8/61.2	73/74	100/100	9/10	20		0.3	2	-	-
OASIS-6 (2007)	2748	61.5/62.1	73.1/71.7	-	14.9/14.0	25	50	0.3	1.5	-	8.5/7.5
IMMEDIATE (2012)	871	63.9/63.3	72.5/69.6	-	29.4/26.3	30	25	0.6	1.5	1.5/1.5	-

*ECLA* = Estudios Cardiologicos Latinoamerica; *POL*-*GIK* = Polish-Glucose-Insulin-Potassium; *GIPS*-I = Glucose–insulin–potassium study-I; *GIPS*-II = Glucose–insulin–potassium study-II; *REVIVAL* = The Reevaluation of Intensified Venous Metabolic Support for Acute Infarct Size Limitation; *OASIS*-6 = Organization to Assess Strategies for Ischemic Syndromes; DIGAMI = the Diabetes and Insulin-Glucose Infusion in Acute Myocardial Infarction; HI-5 = The hyperglycemia: intensive insulin infusion in infarction; IMMEDIATE = the Immediate Myocardial Metabolic Enhancement During Initial Assessment and Treatment in Emergency care Trial.

*numerator/denominator indicates treatment group/control group.

¶median, Glucose (%) and potassium (%) mass concentration.

### Mortality and subgroup analysis

**Figure 2:**
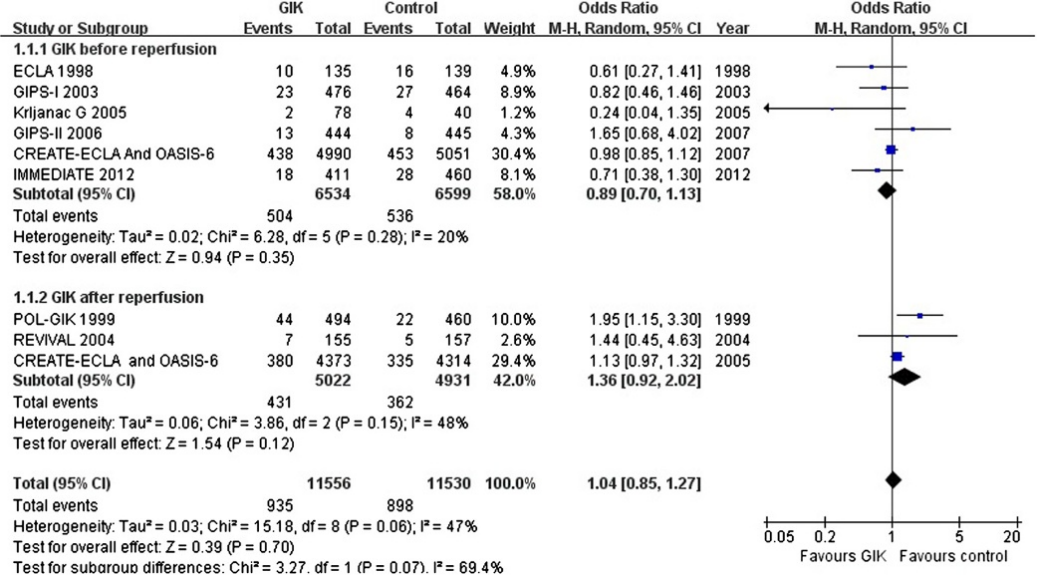
**Forest plot for in**-**hospital mortality according to before or after reperfusion.**

In nine RCTs studying GIK in patients with AMI or ACS, meta analysis did not demonstrate a significant reduction in mortality (OR 1.04, 95% CI 0.85 to 1.27, p = 0.70), although there was still considerable heterogeneity between trials (*I*^*2*^ = 47.0%, *p* = 0.06, Figure 2).

Because the effect of GIK on AMI might vary depending upon the time of GIK treatment (before and after reperfusion), [9] we performed a subgroup analysis to examine the heterogeneity of the results. Five Trial [5, 11, 13, 15, 16] and combined CREATE-ECLA and OASIS-6 subgroup analysis [9] evaluated the effect of receiving GIK before reperfusion and found no significant reduction in mortality (OR 0.89, 95% CI 0.70 to 1.13, *p* = 0.35). The trial by Krljanac et al. [15] showed the largest effect of treatment but it was also the smallest trial. Exclusion of this trial removed the statistical heterogeneity (*I*^*2*^ = 0%, p = 0.43) and did not affect the lack of effect on mortality (OR 0.95; 95% CI 0.84 to 1.03, *p* = 0.447). Two trials [12, 14] and combined CREATE-ECLA and OASIS-6 subgroup analysis [9] studied the effect of GIK after reperfusion. Overall, there was no significant reduction in mortality (OR 1.36, 95% CI 0.92 to 2.02, *p* = 0.12).

**Figure 3:**
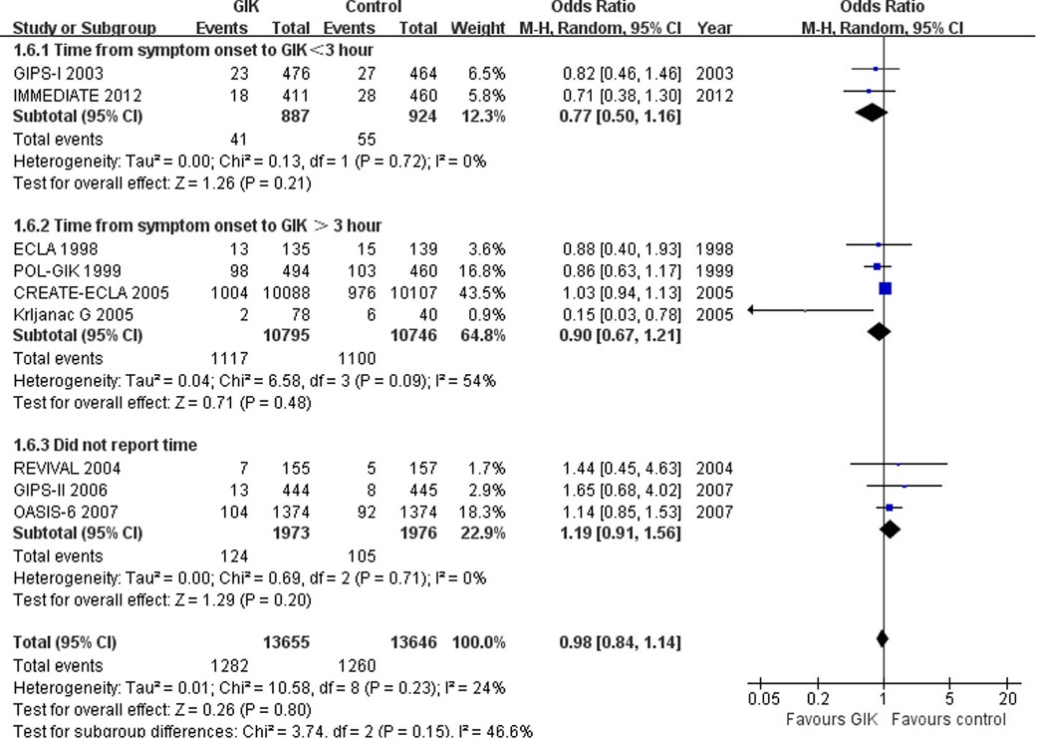
**Forest plot for in**-**hospital mortality according to timeliness from symptom to treatment (<3 hour or >3 hour).**

The potential benefit of GIK is thought to be related to timeliness of administration after onset of cardiac ischemia, so we did subgroup analysis according to time from onset of symptom to treatment (with 3 hours *vs*. >3 hours). Two RCTs [5, 13] evaluated the effect of receiving GIK within 3 hours from onset of symptom to treatment and found no significant reduction in mortality (OR 0.77, 95% CI 0.50 to 1.16, *p* = 0.21). Four trials [4, 11, 12, 15] studied the effect of GIK >3 hours from onset of symptom to treatment. Overall, there was no significant reduction in mortality (OR 0.90; 95% CI, 0.67-1.21, *p* = 0.48) (Figure 3).

### Meta-regression and sensitivity analyses

**Figure 4:**
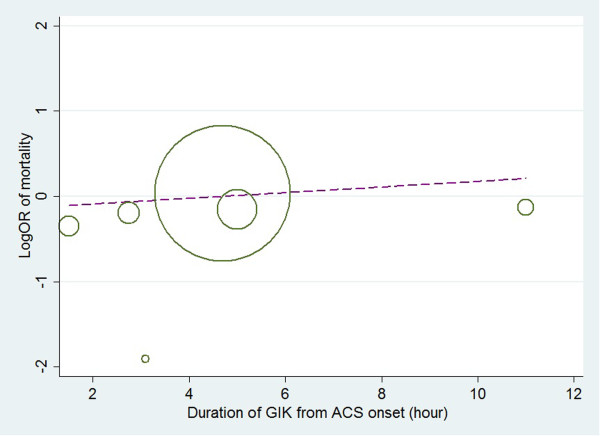
**Meta regression for mortality according to timeliness from symptom to treatment (hours).**

Only six [4, 5, 11–13, 15] of the eligible nine studies reported data on time from onset of symptom to treatment. Meta-regression was used to assess the possible influence of time from onset of symptom to treatment of the six studies [4, 5, 11–13, 15] on the mortality outcome (Figure 4). This analysis showed that heterogeneity could not be explained by time from onset of symptom to treatment (coefficient =0.032, *p* = 0.639).

The two trials showing the largest effect of treatment [11, 15] were also the two smallest, and together accounted for 2% of patients in this analysis. This systematic review did not find any corresponding small negative trials, and thus there is suggestion of publication bias. Although publication bias could result in an overestimation of treatment effect, this would not likely produce a qualitative change in the results of this analysis, given the relatively large number of patients in this analysis. Indeed, a sensitivity analysis, which excluded all reports of <500 patients, still found a significant reduction in mortality, but the apparent treatment effect was considerably smaller (OR: 0.96; 95% CI 0.85 to 1.10, *p* = 0.58).

## Discussion

Our meta-analysis of 9 trials assessing the use of GIK infusions in AMI patients reveals no benefit with these therapies in mortality. Furthermore, subgroup analysis indicates that the GIK is not effective in reducing the mortality of AMI whether reperfusion strategy (before or after reperfusion) or timeliness of symptom to treatment (within 3 hours or >3 hours).

A 2010 meta-analysis by Zhao et al. confirmed that GIK, aiming at the administration of large doses of glucose and insulin without paying attention to an increase in plasma glucose does not improve mortality [18]. The untoward effects of secondary hyperglycemia have been given a plausible explanation. These assumptions were, however, questioned by Selker et al. [9] and Grossman [2] referring to experimental evidence that insulin reduces reperfusion-induced myocar injury. Grossman advocated that to be effective GIK has to be instituted very early after the onset of symptoms indicating myocardial ischemia, which would be the true test of the GIK hypothesis. However, all the studies examined AMI patients ≥3 hours after the onset of symptoms except for Glucose –Insulin - Potassium Study - 1 (GIPS-1) and IMMEDIATE trial.

Further subgroup analysis and meta regression of trials refuted the possibility that GIK would benefit patients when initiated from symptom to treatment within 3 hours, [2] although preclinical studies suggested that GIK could suppress ischemia reperfusion injury [19–21]. There are several possible explanations for the discordance between the positive findings in preclinical studies and the predominantly negative RCTs.

As one interprets the findings of our systematic review, a distinction should be made between the use of GIK and tight glycemic control. The studies reviewed in the present meta-analysis examined the benefit of GIK when administered as metabolic support. That is, these solutions were administered at a set concentration and rate to enhance glucose uptake and utilization within ischemic myocardium. It has been postulated that studies that use GIK as a metabolic ‘cocktail’ were negative because of this lack of attention to glycemic control. It has been established that hyperglycemia at the time of myocardial ischemia is associated with increased mortality in both diabetic and nondiabetic individuals [22]. There is also evidence that acute hyperglycemia is associated with increased platelet and leukocyte activation [23, 24]. It has been speculated that in the CREATE trial, the potential benefits.

In addition, the concomitant medications used during current clinical practice, which are typically absent in experimental studies, may influence the effectiveness of agents directed against reperfusion injury.

### Limitations

The present meta-analysis highlights several shortcomings in the perioperative GIK literature. Many trials examining the use of GIK infusions in AMI are not adequately powered to examine mortality end point. Indeed, mortality was not the primary outcomes in many of the studies analyzed in the present review. In addition, a potential limitation of our meta-analysis is that it was based on trial-specific rather than patient-specific data. Its most important limitation, many trials didn’t report the timeliness from symptom to treatment.

## Conclusions

In summary, this systematic review and meta-analysis showed that administration of GIK in ACS patients does not significantly reduce mortality whether or not GIK administration >3 or <3 hours after the onset of symptoms.

## Electronic supplementary material

Additional file 1: **GRADE summary of evidence for RCTs of GIK in acute coronary syndrome.** (PNG 8 KB)

## Abbreviations

GIK: Glucose-insulin-potassium
ACS: Acute coronary syndrome
OR: Odd ration
CI: Confidence interval
ECLA: Estudios Cardiologicos Latinoamerica
POL-GIK: Polish-Glucose-Insulin-Potassium
GIPS-I: Glucose–insulin–potassium study-I
GIPS-II: Glucose–insulin–potassium study-II
REVIVAL: The Reevaluation of Intensified Venous Metabolic Support for Acute Infarct Size Limitation
OASIS-6: Organization to Assess Strategies for Ischemic Syndromes
DIGAMI: The Diabetes and Insulin-Glucose Infusion in Acute Myocardial Infarction
HI-5: The hyperglycemia: intensive insulin infusion in infarction
IMMEDIATE: The Immediate Myocardial Metabolic Enhancement During Initial Assessment and Treatment in Emergency care Trial
PRISMA: Preferred reporting items for systematic reviews and meta-analyses guidelines
RCT: Randomized controlled trial.
