# Supplementary figures and images for: Glucose–insulin–potassium therapy in patients with acute coronary syndrome: a meta-analysis of randomized controlled trials
Source: BMC Cardiovasc Disord. 2014 Nov 25;14:169. doi: 10.1186/1471-2261-14-169 (PMC4256054; doi:10.1186/1471-2261-14-169)

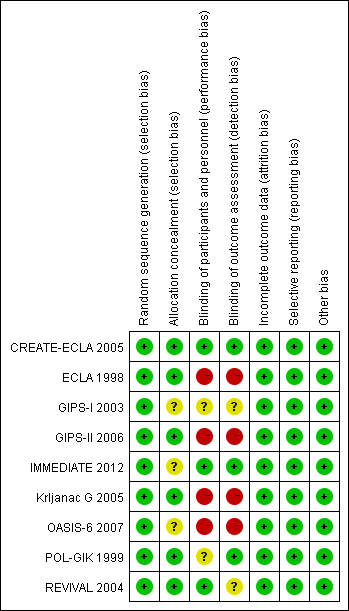

Supplement: Supplementary file 1 — Additional file 1: GRADE summary of evidence for RCTs of GIK in acute coronary syndrome. (PNG 8 KB) [file 12872_2014_812_MOESM1_ESM.png]
